# Temperature-dependent developmental modeling of protophormia terraenovae (Diptera: Calliphoridae) and its application in PMI inference

**DOI:** 10.3389/finsc.2026.1774730

**Published:** 2026-02-18

**Authors:** Yali Guo, Yuequn Niu, Bo Wang, Zhou Li, Minghao Zhang, JiaHao Guo, Jifeng Cai, Fanming Meng

**Affiliations:** 1 Department of Forensic Medicine, School of Basic Medical Sciences, Xinjiang Medical University, Urumqi, Xinjiang, China; 2 Key Laboratory of Forensic Medicine, Xinjiang Medical University, Urumqi, China; 3 Department of Forensic Science, School of Basic Medical Sciences, Central South University, Changsha, Hunan, China; 4 Department of Parasitology, Central South University, Changsha, China

**Keywords:** forensic entomology, necrophagous insect, *Protophormia terraenovae*, postmortem interval, developmental models

## Abstract

*Protophormia terraenovae* is a forensically important blow fly species in cold regions. This study investigated its development at constant temperatures (15-25°C). Results showed that developmental duration significantly decreased with increasing temperature, with the total period ranging from 779.33 hours at 15 °C to 396.67 hours at 25 °C. The hatching and third-instar larval stages were most temperature-sensitive. We established Isomorphen and Isomegalen models, which visually illustrated the prolongation of development progress and the increased time required for larval growth per millimeter as temperature decreased. Thermal summation models indicated a strong linear relationship for the hatching and third-instar stages. Furthermore, we found that pupal weight was a more reliable growth indicators than length or width. This study provides fundamental developmental data and models for improving the accuracy of postmortem interval estimation using *P. terraenovae* in forensic practice.

## 1 Introduction

Estimating the postmortem interval (PMI) based on the developmental rate of necrophagous flies is a cornerstone of modern forensic entomology (1). The accuracy of this estimation largely depends on developmental models established from constant-temperature experiments. Temperature is the primary abiotic factor driving these developmental rates, and accurate, species-specific thermal models are the prerequisite for any reliable forensic inference. Therefore, studying insect growth data under different temperatures is of great significance for PMI estimation.

*Protophormia terraenovae* (Robineau-Desvoidy, 1830)(Diptera: Calliphoridae) is known to be a primary colonizer during the early stages of human decomposition in the field of forensic entomology (2). It is distributed both domestically and internationally, widely found in cold regions of Europe and North America (3). In Europe, it is widely distributed from the Arctic to subtropical regions, including Spain, the Czech Republic, and Slovakia (4–6). In North America, it includes the United States and Canada (7, 8). In China, it is mainly distributed in Tibet, Qinghai, Xinjiang, Heilongjiang, Sichuan, and other regions (9–11). Most regions exhibit significant seasonal temperature differences, characterized by long, cold winters and warm or even hot summers,with relatively limited precipitation. Unlike thermophilic species that thrive in mid-summer, *P. terraenovae* is highly cold-tolerant and frequently appears as a primary colonizer in early spring, late autumn, or cool-temperate regions (12). In many cold-weather cases, it serves as the sole biological evidence available to investigators (11). Therefore, establishing a precise developmental timeline for this species is essential for solving cases in low-temperature environments globally.

Although the developmental progress of *P. terraenovae* has been documented in previous studies (6, 13, 14), there are still deficiencies in the application of developmental models and the accuracy of PMI estimation in later developmental stages. Many existing studies only provide basic development timelines (6) but lack comprehensive Isomorphen diagram, Isomegalen diagram, and Thermal summation models, which are crucial for rapid visual estimation in cases (14). More importantly, a major challenge in forensic entomology is the PMI estimation of the pupal stage. The pupal stage typically constitutes a significant portion of the larval life cycle, but morphological changes during this period are subtle and difficult to quantify (15). Traditional length measurements show almost no change after pupation, leading to a blind spot in PMI estimation. Therefore, there is an urgent need to explore alternative indicators, such as dynamic weight changes during the pupal stage, to accurately determine the age of this stage.

To address these limitations, this study systematically investigates the temperature-dependent developmental progress of *P. terraenovae* under constant temperatures ranging from 15 to 25°C. The objectives of this study are: (1) to provide a dataset on developmental duration for this cold-tolerant species; (2) to construct robust Isomorphen diagram, Isomegalen diagram, and Thermal summation models to visualize growth patterns; (3) to evaluate the reliability of pupal morphological changes as an auxiliary indicator for PMI estimation. By integrating these multidimensional models, this study aims to enhance the accuracy of inferring PMI using the developmental status of *P. terraenovae*, providing more comprehensive tools for forensic practitioners in cold regions.

## 2 Materials and methods

### 2.1 Insect source and colony establishment

Adult *P. terraenovae* were collected from sheep carcasses in Qitai County, Xinjiang, China (44.01°N, 89.59°E). Adult flies were identified morphologically under a stereomicroscope (16). One male and one female were paired to establish laboratory colonies. Larvae were reared on fresh pork lung, and colonies were maintained for at least three generations before experiments. Adults were kept in nylon mesh cages (50 × 50 × 50 cm) at 25 ± 1°C under natural photoperiod.

### 2.2 Experimental design

Eggs laid within 2 hours were randomly distributed across three constant-temperature regimes (15, 20 and 25°C). Each treatment consisted of three biological replicates, and each replicate included at least 100 eggs from a different egg mass. Relative humidity was maintained at 75% with a 12 h:12 h (L:D) photoperiod.

### 2.3 Observation of developmental duration and measurement of larval morphological indicators

Egg hatching was monitored at 2-hour intervals until all eggs had hatched. Growth indicators was recorded at 12-hour intervals. Five larvae were randomly sampled at each time point until pupariation for body length measurement and instar identification. The instar was determined based on the number of spiracular slits in the posterior spiracles: two slits indicated the second instar larval, and three slits indicated the third instar larval (17). Upon sampling, larvae were killed in hot water (>90 °C) for five minutes (18) and preserved in 1.5 ml of 70% ethanol in EP tubes (19). Body length and body width were measured using a digital vernier caliper with an accuracy of 0.01 mm and (20). After pupariation, developmental status was checked every 24 hours until adult eclosion.

### 2.4 Experimental data analysis

All experimental data were first preliminarily organized using Excel. Statistical analysis was performed with Origin Pro 2021 to calculate the mean and standard deviation (SD) of each indicator. It was also used to complete the fitting and visualization of all models, generating model curves and 95% confidence intervals. One-way ANOVA was employed to test the significance of differences in developmental duration and morphological indicators among different temperature treatment groups, with Tukey’s method used for multiple comparisons, and the significance level set at α=0.05.

## 3 Results and analysis

### 3.1 Isomorphen models are based on development

Different temperatures have significant effects on the duration of growth and development stages of *P. terraenovae* (**Table 1**). One-way ANOVA results indicate that within the 15-25 °C temperature range, developmental stages including hatching, second instar, third instar, and pupation all showed prolonged durations with decreasing temperatures, with the total developmental period also increasing. When the temperature dropped from 25 °C to 15 °C, the total developmental period extended from 396.67 ± 13.58 hours to 779.33 ± 36.23 hours (F = 72.58, P<0.001). Statistical significance results showed no significant differences in the wandering and eclosion stage under different temperature conditions (wandering: F = 1.4091, P = 0.3150>0.05; eclosion: F = 1.2895, P = 0.3421>0.05). However, significant differences were found in the hatching stage(F = 16.9821, P = 0.0034<0.05), third instar larval stage (F = 24.3636, P = 0.0013<0.01), and total development duration (F = 72.5798, P = 0.0000<0.001). Furthermore, the eclosion rates at 15 °C, 20 °C and 25 °C were 88.72%, 76.63% and 77.78%, with a slightly higher eclosion rate under lower temperature conditions.

**Table 1:** Developmental duration of different stages at different temperatures.

Developmental stage	Temperature treatment group			F	P	R²
	25°C	20°C	15°C			
Hatching	20.67 ± 0.58^b^	43.00 ± 0.00^b^	79.33 ± 21.55^a^	16.9821	0.0034	0.8499
2nd instar	64.00 ± 6.93^ab^	52.00 ± 6.92^b^	84.00 ± 12.00^a^	9.8000	0.0129	0.7656
3rd instar	52.00 ± 13.86^b^	84.00 ± 31.75^b^	196 ± 30.20^a^	24.3636	0.0013	0.8904
Wandering	36.00 ± 31.75^a^	40.00 ± 6.93^a^	60.00 ± 31.75^a^	1.4091	0.3150	0.31960
Pupation	160.00 ± 13.86^b^	172.00 ± 6.93^b^	264.00 ± 31.75^a^	23.3462	0.0015	0.8861
Eclosion	64.00 ± 18.34^a^	76.00 ± 36.66^a^	96.00 ± 12.00^a^	1.2895	0.3421	0.3006
Total	396.67 ± 13.58^b^	467.00 ± 60.40^b^	779.33 ± 36.23^a^	72.5798	6.25383E-5	0.9603
Eclosion rate	77.78%	76.63%	88.72%	–	–	–

Values are mean ± SD. Means followed by different letters are significantly different at P < 0.05 (Tukey’s test). The R^2^, F- and P-values from one-way ANOVA are shown; P < 0.05 and P < 0.01 represent significant and highly significant differences, respectively.

**Figure 1:**
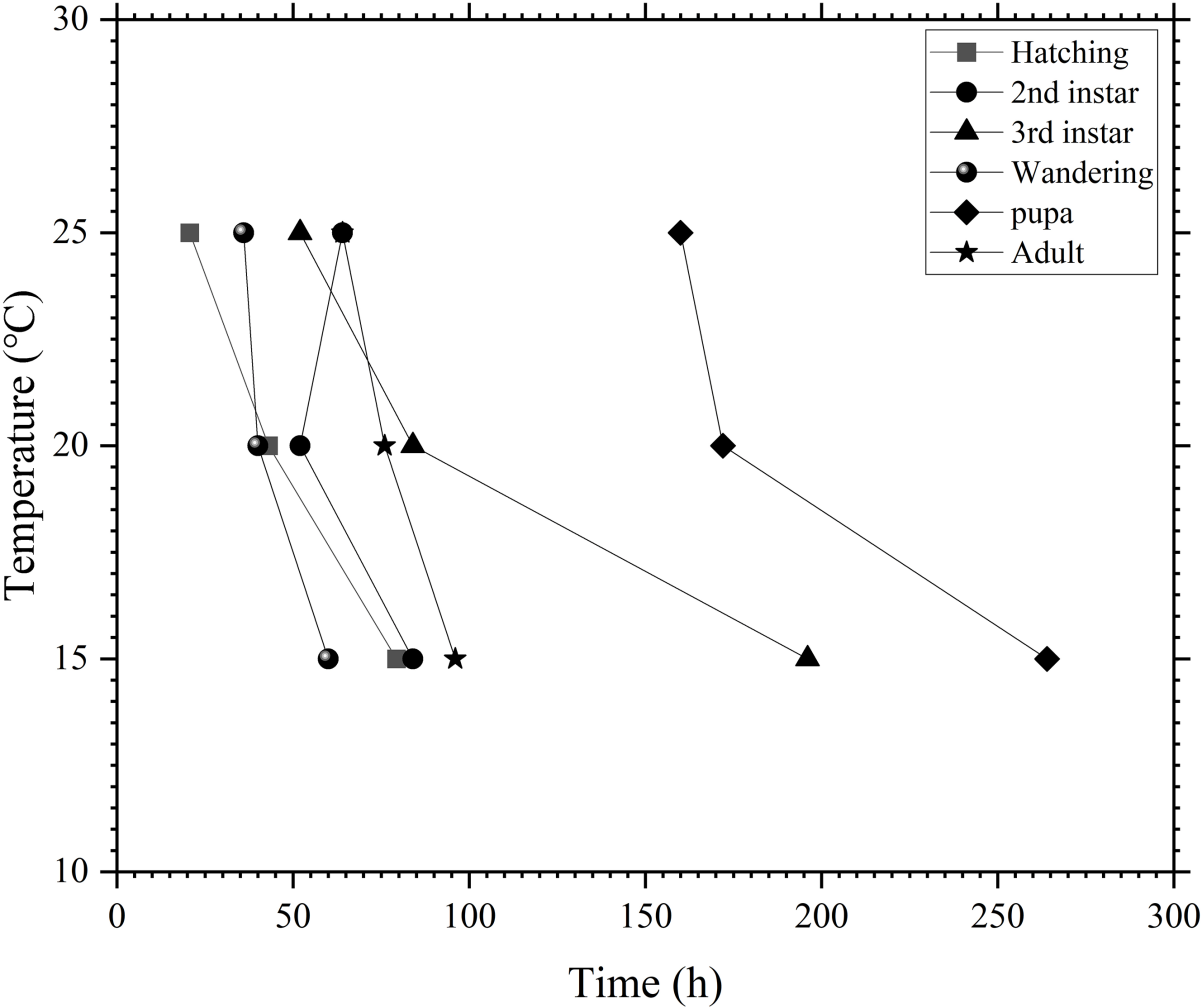
Isomorphen diagram of *P. terraenovae* development at constant temperatures.

The Isomorphen diagram for *P.terraenovae* was plotted using OriginPro 2021, with the time to reach each developmental milestone as the x-axis and temperature as the y-axis. As shown in the diagram (**Figure 1**), within the temperature range of 15-25 °C, the time required to complete each developmental milestone gradually increased as the temperature decreased. Moreover, the spacing between successive curves widened with decreasing temperature, reflecting a clear slowdown in the developmental rate under lower temperatures.

### 3.2 Isomegalen models are base on growth

**Figure 2:**
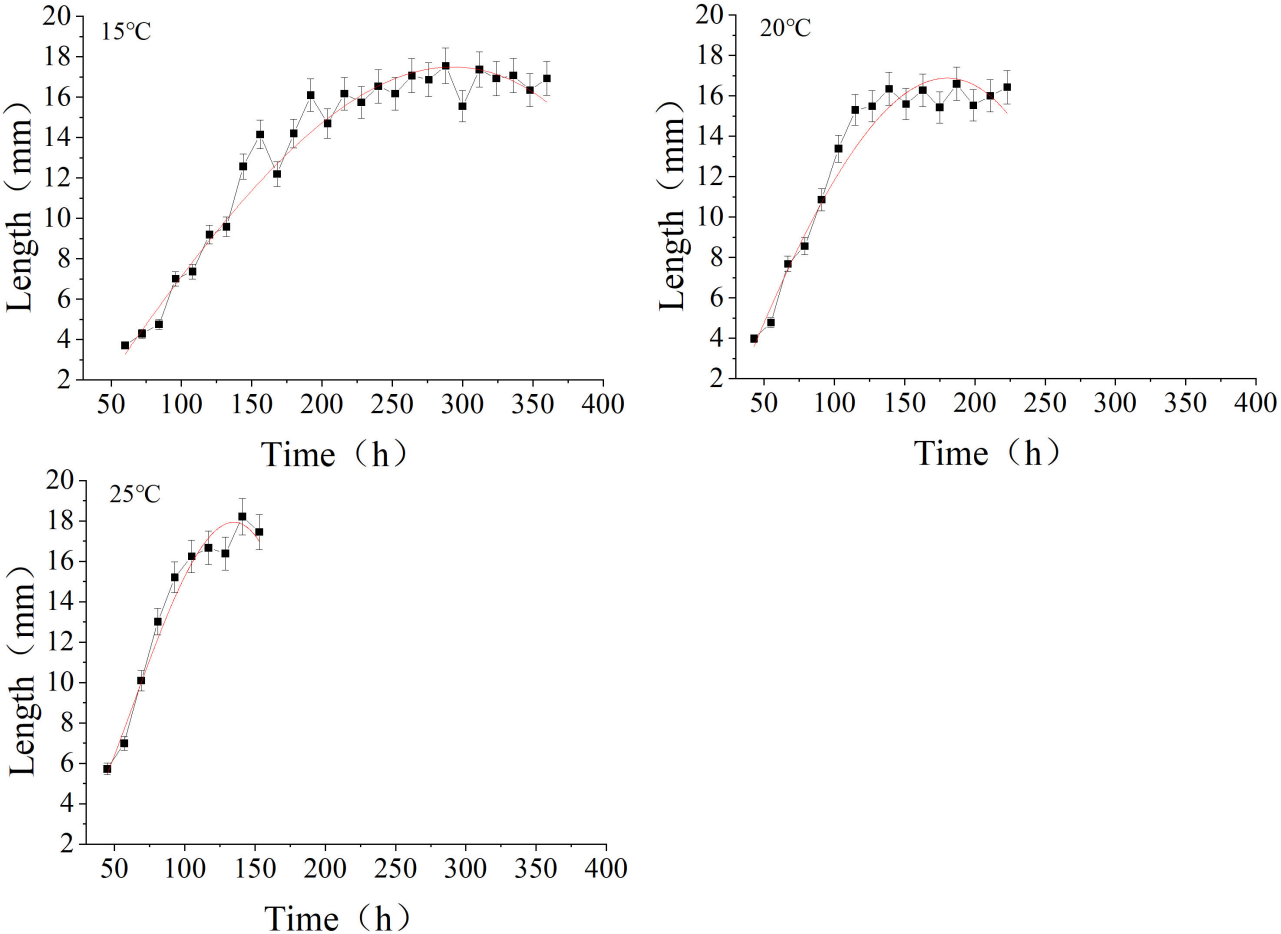
Simulated curves depicting the change in larval body length over time at different temperatures. Data points (■) represent the mean larval body length, with error bars indicating the standard deviation.

To clarify the developmental progress of *P.terraenovae* larvae under constant temperatures of 15 °C, 20 °C and 25 °C, regression analysis was performed with the time after larva hatched as the independent variable and larval body length as the dependent variable.The analysis yielded simulated curves (**Figure 2**) and corresponding equations (**Table 2**) that describe the changes in larval body length over time at each temperature. The analysis of the curve morphology showed an S-shaped trend, characterized by a rapid increase followed by a plateau under all three temperature conditions. At 25 °C, the initial growth rate of larval body length was the most rapid among the three temperature groups, while the slowest growth occurred at 15 °C. The simulation equations derived from nonlinear fitting yielded R² values (15 °C: 0.9800, 20 °C: 0.9777, 25 °C: 0.9823). All values were close to 1, indicating an excellent model fit. This demonstrates that the model reliably predicts larval body length based on developmental duration of different temperatures, thereby validating the use of body length as an auxiliary indicator for PMI estimation.

**Table 2:** Simulation equation depicting the change in larval body length over time at different temperatures.

Temperature	Simulation equation	R^2^
15 °C	L=(-2.83809 ± 1.40596) + (0.10291 ± 0.02978)T + (8.35363E-6 ± 1.75276E −4)T^2^ + (−4.18914E−7 ± 3.02249E−7)T^3^	0.9800
20 °C	L=(-4.02386 ± 2.30086) + (0.18566 ± 0.07408)T + (-1.30549E-4 ± 6.75728E-4)T^2^ + (-1.42089E-6 ± 1.82613E-6)T^3^	0.9777
25 °C	L=(0.17901 ± 5.84615) + (0.04756 ± 0.21955)T + (0.00228 ± 0.00252)T^2^ + (-1.21548E-5 ± 8.92779E-6)T^3^	0.9823

**Figure 3:**
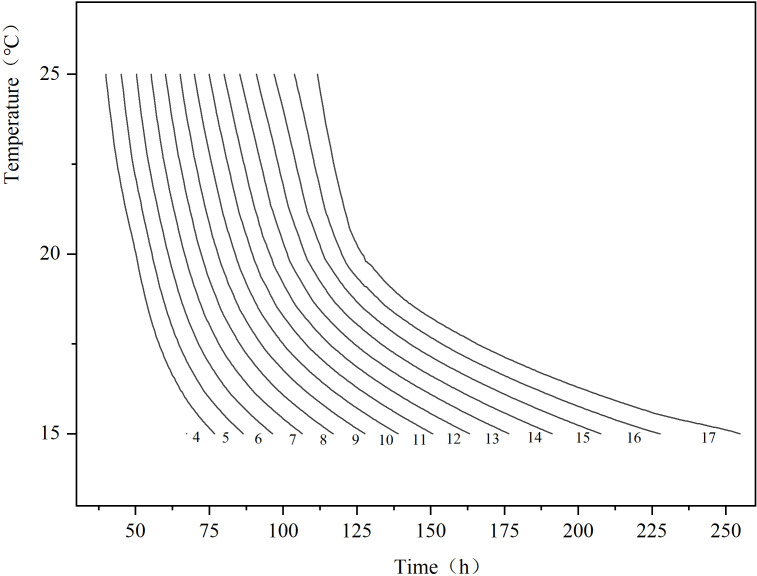
Isomegalen diagram of *P.terraenovae* larvae from hatching to peak feeding stage. Time was plotted against temperature where each line represents developmental larval length in 4–17 mm, size is indicated by the number at the lower margin of each contour.

Based on the larval body length change curves and simulation equation data from **Figure 2**; **Table 2**, an Isomegalen diagram was established (**Figure 3**). This model can be used to estimate larval age. The model shows that as the temperature decreases, the distance between the contour lines gradually widens. It indicates that the developmental time for growth by 1 mm is correspondingly prolonged.

### 3.3 Thermal summation model

**Figure 4:**
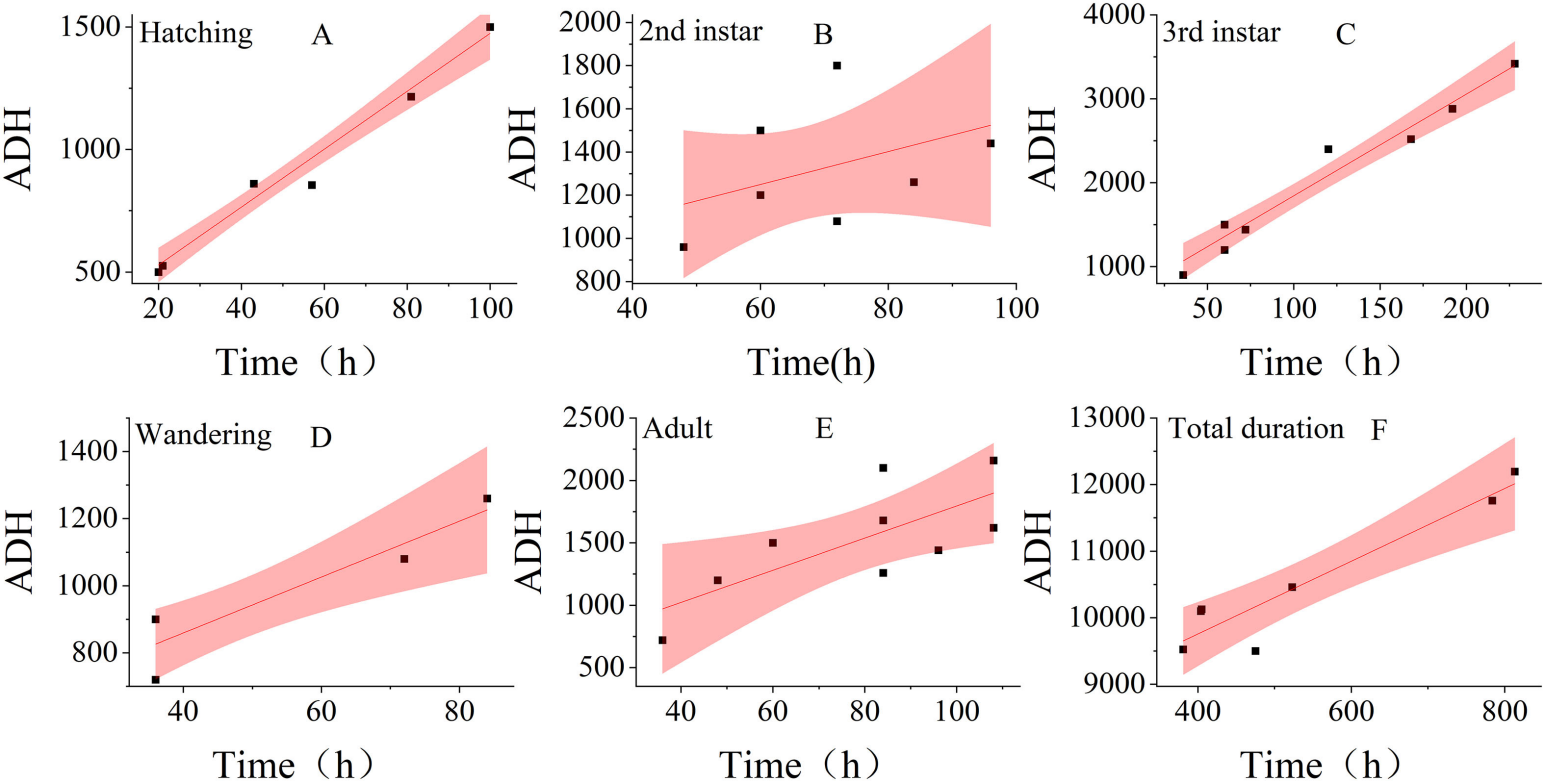
Thermal summation model of five developmental stages and a total developmental stage. The red shaded area indicates the 95% confidence interval. **(A–F)**: Hatching, second instar, third instar, Wandering, Eclosion, and total duration.

**Figure 5:**
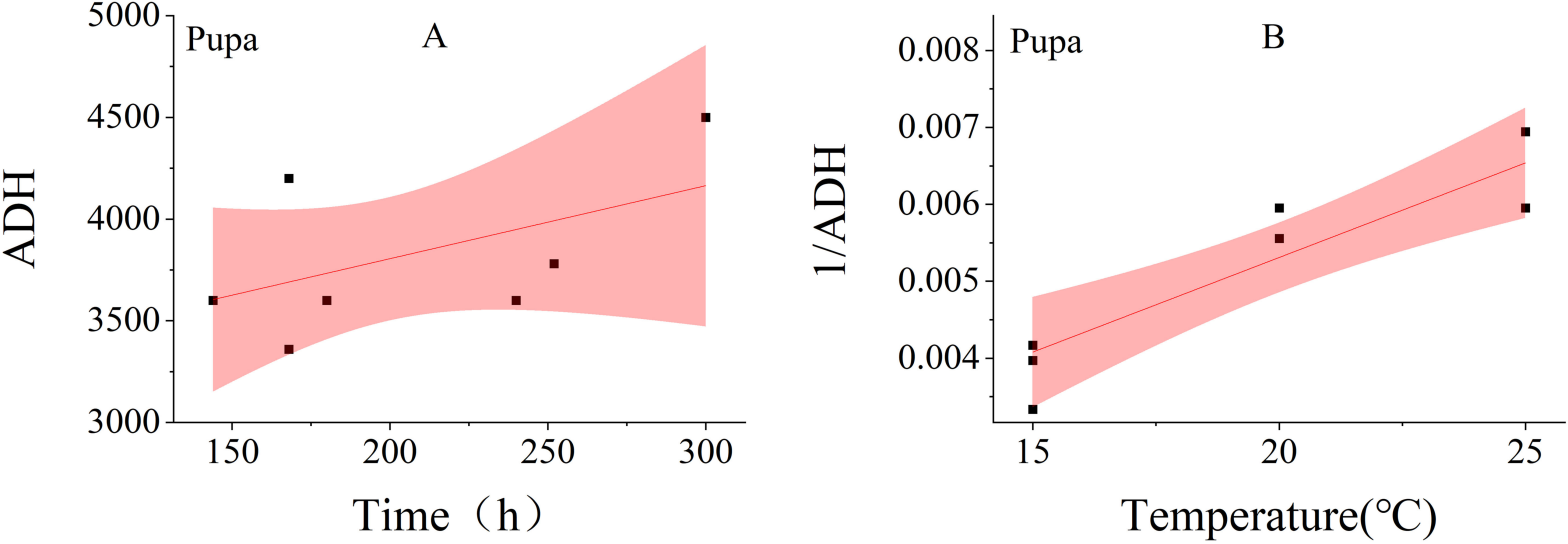
Thermal summation model of pupation stage of different fitting methods. linearized equation [**(A)** D(T−t)=k, **(B)** $\frac{1}{D}=-\frac{t}{k}+\frac{1}{k}T$].

Linear regression analysis was performed using the duration of each developmental stage—hatching, second instar, third instar, wandering, eclosion and total developmental period—as the independent variable (X-axis) and the accumulated degree hours (Chattopadhyay et al.) as the dependent variable (Y-axis). This resulted in the establishment of six linear thermal summation models (**Figure 4**). The results showed that most of the data points for stages such as hatching and the third instar stage were distributed within the confidence intervals, and the linear fitting degree was good, demonstrating the reliability of the linear regression models for these stages. However, the fitting effect of the model for the pupation stage was poor when the ADH was used as the Y-axis, and the fitting effect was better when the developmental rate (1/ADH) was used as the Y-axis (**Figure 5**). Due to the poor linear correlation between effective thermal summation (K) and developmental time for the total developmental period at 20 °C and 15 °C, as well as for the pupariation and post-feeding stages at 25 °C and 15 °C, these data points were excluded from the regression analysis following the recommendation of Ikemoto and Takai (21).

The lower temperature threshold (D_0_) and the effective cumulative temperature (K) for each stage were obtained from the effective thermal summation model (**Table 3**). The model fit was highly satisfactory for the eclosion and third-instar stages, with R² values of 0.9707 and 0.9618, respectively, demonstrating a strong linear relationship. Conversely, the low R² values of 0.1854 for the second instar stage and 0.2146 for pupariation indicated a weak linear relationship in these stages.

**Table 3:** Lower temperature threshold (D_0_) and effective cumulative temperature(K) at different developmental stages.

Development stage	K ± SD	D_0_ ± SD	R2
Hatching	292.59092 ± 42.22426	11.81045 ± 0.77611	0.9707
2nd instar	792.1875 ± 412.44018	7.61719 ± 6.03406	0.1854
3rd instar	632.74086 ± 117.33543	12.11379 ± 0.91268	0.9618
Wandering	525.71429 ± 93.28473	8.33333 ± 1.80388	0.8102
Pupation	3086.54015 ± 531.62711	3.59124 ± 2.59704	0.2146
Eclosion	506.50307 ± 368.5154	12.88344 ± 4.47946	0.5417
Total duration	7569.56127 ± 481.11602	5.46316 ± 0.84914	0.8922

### 3.4 Changes in pupal growth indicators

**Figure 6:**
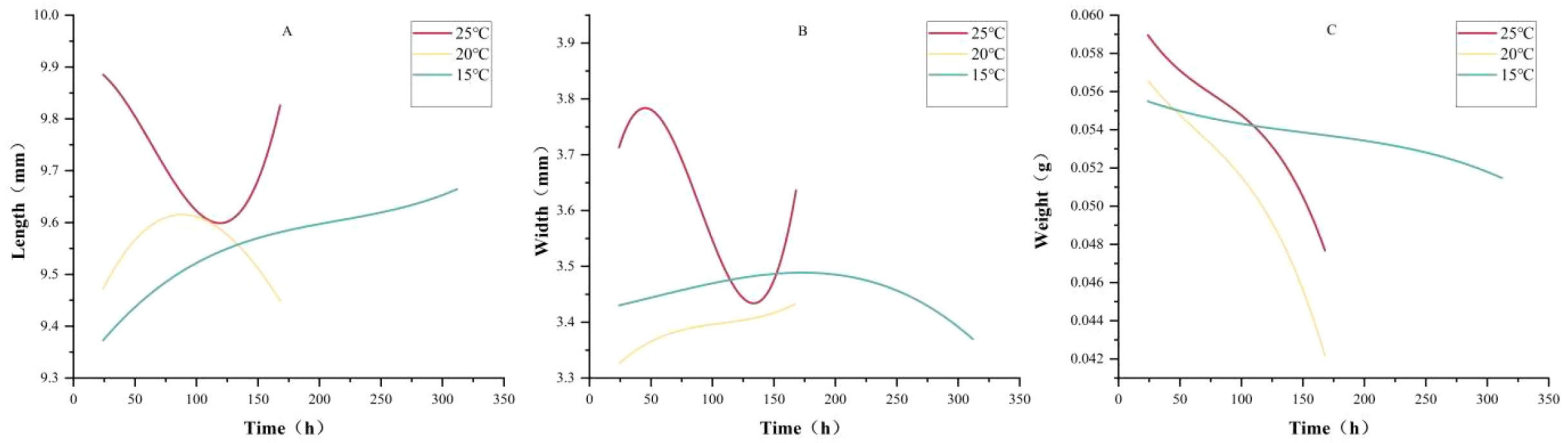
Simulated curves of pupal growth indicators changes over time at different temperatures. X: developmental time (hours), Y: morphological measurements [**(A)** body length; **(B)** body width; **(C)** body weight].

This study further conducted dynamic monitoring of changes in body length, width, and weight during the pupal stage of *P. terraenovae*, establishing corresponding cubic polynomial regression models (**Figure 6**) and simulation equations (**Table 4** Simulated equations for pupal growth indicators changes over developmental time at different temperatures). The simulated curves revealed considerable variation in the trends of pupal body length and width across the three temperatures, with no consistent pattern observed. In contrast, the pupal weight curves exhibited a gradual declining trend under all temperature conditions, with the rate of weight loss accelerating as the pupal stage approached eclosion. The weight change was slowest and showed the least curve fluctuation at 15 °C. Analysis of the R² values from the simulation equations indicated a high goodness-of-fit for the weight change curves at 25 °C (R² = 0.9203) and 20 °C (R² = 0.957). However, the fit was poor at 15 °C (R² = 0.2897), suggesting that low temperatures significantly disrupt the normal pattern of pupal weight change. At 25 °C and 20 °C, body weight has a relatively higher fit compared to body length and body width, suggesting that the weight may better reflect the physiological state of pupal development and could be used for further model analysis. Under extremely low temperature conditions, body weight may not be a good indicator of growth.

**Table 4:** Simulated equations for pupal growth indicators changes over developmental time at different temperatures.

Temperature	Growth indicators	Simulated equations	R^2^
25°C	Length (mm)	L=(0.0012 ± 0.01427)T + (-8.35138E-5 ± 1.67244E-4)T^2^ + (4.39393E-7 ± 5.75591E-7)T^3^	0.6407
20°C		L=(0.00684 ± 0.01502)T +(-4.65282E-5 ± 1.76042E-4)T^2^ + (5.89353E-8 ± 6.05871E-7)T^3^	0.3577
15°C		L=(0.00341 ± 0.00581)T + (-1.39097E-5 ± 3.94372E-5)T^2^ + (2.15819E-8 ± 7.73515E-8)T^3^	0.2951
25°C	Width (mm)	W=(0.01845 ± 0.01202)T + (-2.72983E-4 ± 1.40881E-4)T^2^ + (1.01809E-6 ± 4.84861E-7)T^3^	0.7765
20°C		W=(0.00311 ± 0.00418)T + (-2.60554E-5 ± 4.90051E-5)T^2^ + (8.03755E-8 ± 1.68657E-7)T^3^	0.6786
15°C		W=(4.39334E-4 ± 0.00315)T + (1.93412E-6 ± 2.13776E-5)T^2^ + (-1.23374E-8 ± 4.19298E-8)T^3^	0.1904
25°C	Weight (g)	W=(-1.43017E-4 ± 1.93901E-4)T + (1.29382E-6 ± 2.27207E-6)T^2^ + (-5.59615E-9 ± 7.81963E-9)T^3^	0.9203
20°C		W=(-1.14275E-4 ± 1.84701E-4)T + (8.80181E-7 ± 2.16427E-6)T^2^ + (-4.71536E-9 ± 7.44863E-9)T^3^	0.9567
15°C		W=(-2.77036E-5 ± 7.92385E-5)T + (1.26933E-7 ± 5.37723E-7)T^2^ + (-2.74288E-10 ± 1.05468E-9)T^3^	0.2896

## 4 Discussion

This study systematically observed the developmental progress of *P. terraenovae* under constant temperatures of 15 °C, 20 °C, and 25 °C. The results showed that total development time from egg to adult significantly shortened with increasing temperature, exhibiting a typical temperature-dependent developmental pattern, which is consistent with the findings of Martínez-Sánchez et al. (6). This further indicates that temperature is the core environmental factor driving the developmental rate of this species. It is worth noting that the eclosion rate of the 15 °C group was higher than those of 20 °C and 25 °C groups, suggesting that this species canmaintains a relatively good survival ability at lower temperatures. In light of its ecological context of distribution in cold regions and dominance during early spring and late autumn, this result supports the cold tolerance characteristics of *P. terraenovae* at the level of biological performance. The potential mechanism may be attributed to energy redistribution at low temperatures (22, 23) and accumulating cryoprotectants such as glycerol, glycogen and antifreeze protein to avoid damage from low temperatures (24). However, these inferences still require further verification through subsequent physiological and molecular experiments.

There are significant differences in temperature sensitivity across different developmental stages. In this study, the hatching period and the third-instar larval period exhibited the strongest response to temperature. This may be because the former is a critical physiological node during the transition from egg to larva, while the latter is the main feeding and rapid weight-gain stage, during which physiological metabolism are more sensitive to temperature (25). However, the wandering and enclosion stage showed no significant differences among the temperature treatments, which may be related to the metabolic rate in the non-feeding stages (26). Thses stage rely more on nutrient reserves accumulated during the larval stage for development, with metabolic activities shifting toward tissue remodeling, resulting in significantly reduced temperature dependence (27). This phenomenon is also consistent with the results observed by Day et al. (28) who found that the wandering larval behavior and body length changes of *Calliphora augur* Fabricius were unaffected by temperature. Therefore, when inferring PMI, attention should be paid to the time information provided by temperature-sensitive stages.

Analysis of growth indicators revealed that larval body length increases in an S-shaped curve with development time and has a high degree of fit, indicating that body length can still be used as an auxiliary quantitative indicator for estimating PMI during the larval stage under constant temperature conditions. This conclusion is consistent with the research direction of temperature-length models for various necrophilic insects. For example, in genera such as Calliphora, Lucilia, and Chrysomya, the length-time curves during the larval feeding stage have good age-indicative properties and are often combined with the isomegalen model for PMI estimation. By comparison, the pupal stage exhibits a “weight-priority” characteristic. At 25 °C and 20 °C, the R² values for the pupal weight higher than length and width. This discrepancy is likely due to the pupal case restricting dynamic changes in external dimensions, while weight directly reflects internal metabolic consumption (such as water loss and fat decomposition), making it a more accurate representation of physiological development (29). However, at 15°C, the fitting of the three pupal stage growth indicators decreased. This may be due to reduced enzymatic catalytic efficiency and cell division rates at low temperatures (30), which interfere with normal physiological processes during the pupal stage indicating that low temperature may disturb normal pupal physiological processes, hereby increasing the uncertainty in PMI estimation during the pupal stage. This trend is consistent with reports of developmental disruption in the pupal stage under low temperatures in species such as *Anastrepha grandis* (Macquart, 1846), *Drosophila subobscura* (Collin, 1936) and *Lucilia cuprina* (Wiedemann, 1830) (31–34). Although biological replicate samples were used and the sample size for each replicate (100 eggs) was sufficient, the high variability in the pupation stage may affect the model’s accuracy. This suggests that it is more prudent to prioritize larval development data in cold-temperature cases.

**Figure 7:**
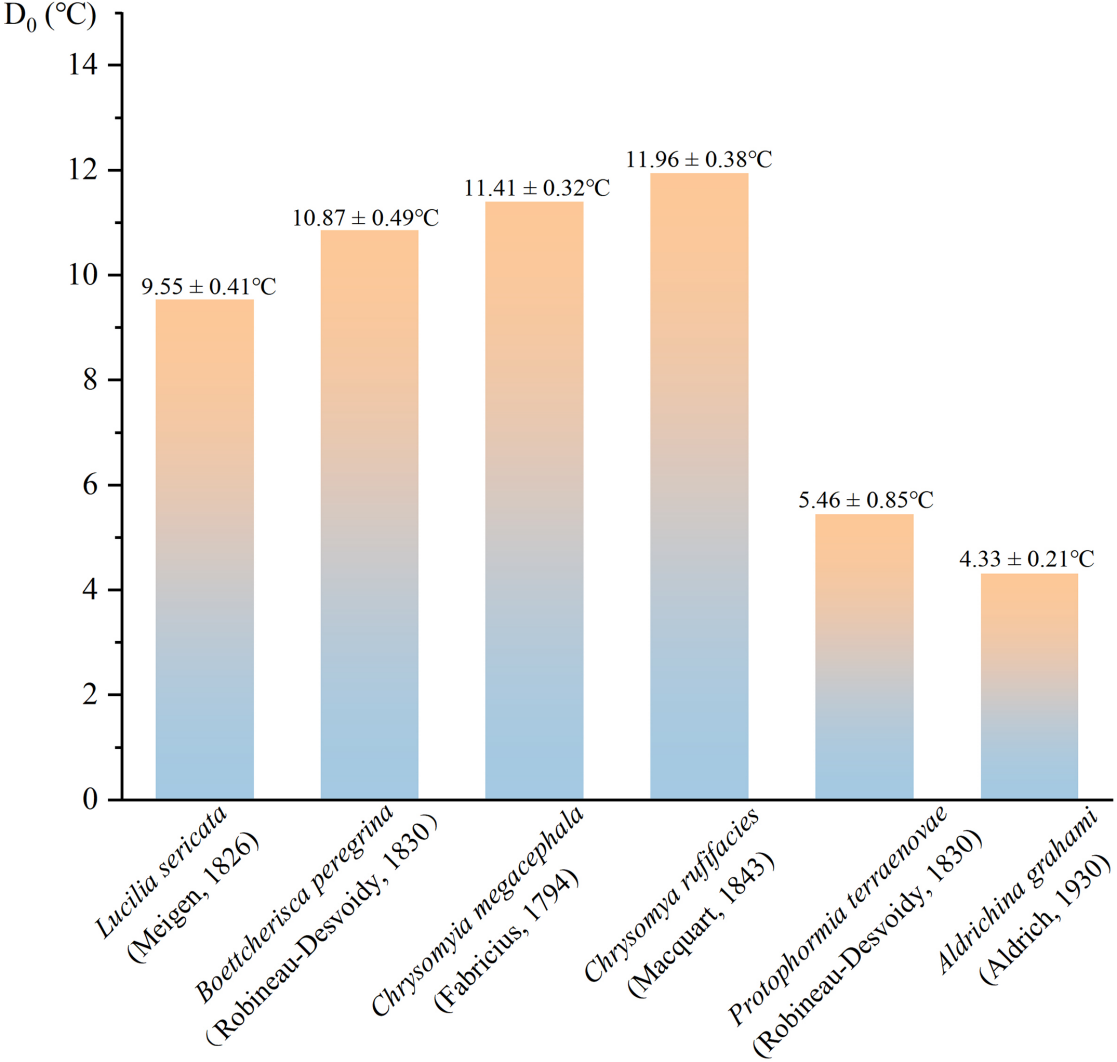
Developmental threshold temperatures (D_0_, mean ± SD) of six common necrophilic flies species. Values represent the minimum effective developmental temperature for each species. Data for *P. terraenovae* are from the present study; data for all other species are sourced from cited literature.

To more intuitively demonstrate the low-temperature developmental advantage of *P. terraenovae*, its total developmental lower temperature threshold (D_0_) is compared with that of common necrophilic flies (35–40). These values are derived from published development model studies. As shown in (**Figure 7**), the total developmental threshold of *P. terraenovae* is significantly lower than that of several typical warm-season dominant species. Simultaneously, like the cold-season dominant species *Aldrichina. Grahami* (Aldrich, 1930), it also has a low developmental lower limit. In addition, our study also found that this fly can complete its life cycle at 10 °C and survive for more than one month at 5°C, although the hatching rate is extremely low. In terms of eclosion rate, *P. terraenovae* has a higher eclosion rate at 15°C than *Lucilia sericata* (Meigen, 1826) (35) and our experiments also found that the eclosion rate is highest at 15°C. These findings collectively indicate that *P. terraenovae* is more likely to become the dominant pioneer species in cold or early late autumn environments. Consequently, it holds significant value for PMI estimation in low-temperature scenarios.

This study also has certain limitations. The experiment was conducted only under constant temperature conditions, failing to simulate temperature fluctuations in natural environments. The temperature gradient setting did not cover the lower temperature range that the species might tolerate. Future research could be carried out under a wider low-temperature gradient (e.g., 5-12 °C) and diurnal temperature fluctuation conditions to establish a growth and development model more closely resembling natural environments. Meanwhile, physiological and biochemical assays combined with molecular biological methods should be employed to deeply elucidate its cold tolerance mechanisms, thereby further improving the application data for PMI estimation in cold regions.

## 5 Conclusion

This study systematically constructed growth data of *P. terraenovae* under constant temperature conditions of 15-25 °C, and successfully established visualizable Isomorphen models, Isomegalen models, and effective accumulated temperature models applicable to forensic practice. The research results clarify the temperature-dependent characteristics of the species, reveal the potential of pupal weight changes as an indicator for PMI estimation, and confirm its developmental advantage in low-temperature environments. These models and basic data provide direct and reliable tools to improve the accuracy of PMI estimation based on *P. terraenovae* in cold regions. Future research should focus on model validation under variable temperature conditions, exploration of lower temperature thresholds, and deeper analysis of cold tolerance mechanisms to promote the application and development of forensic entomology in complex environments.

## Data Availability

The original contributions presented in the study are included in the article/supplementary material. Further inquiries can be directed to the corresponding authors.
